# Resolving Chemical Dynamics in Biological Energy Conversion: Long-Range Proton-Coupled Electron Transfer in Respiratory Complex I

**DOI:** 10.1021/acs.accounts.1c00524

**Published:** 2021-12-13

**Authors:** Ville R. I. Kaila

**Affiliations:** Department of Biochemistry and Biophysics, Stockholm University, 10691 Stockholm, Sweden

## Abstract

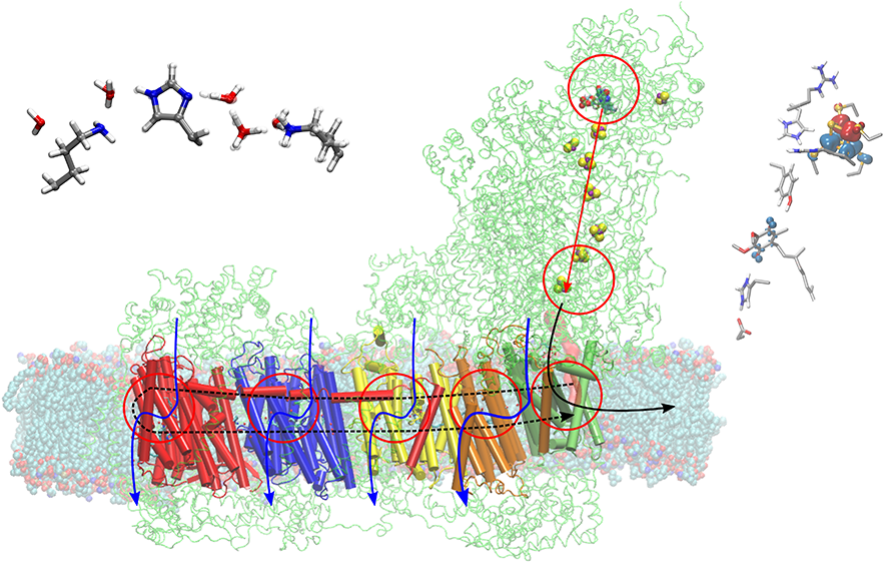

Biological energy conversion is catalyzed by membrane-bound proteins that transduce chemical or light energy into energy forms that power endergonic processes in the cell. At a molecular level, these catalytic processes involve elementary electron-, proton-, charge-, and energy-transfer reactions that take place in the intricate molecular machineries of cell respiration and photosynthesis. Recent developments in structural biology, particularly cryo-electron microscopy (cryoEM), have resolved the molecular architecture of several energy transducing proteins, but detailed mechanistic principles of their charge transfer reactions still remain poorly understood and a major challenge for modern biochemical research. To this end, multiscale molecular simulations provide a powerful approach to probe mechanistic principles on a broad range of time scales (femtoseconds to milliseconds) and spatial resolutions (10^1^–10^6^ atoms), although technical challenges also require balancing between the computational accuracy, cost, and approximations introduced within the model. Here we discuss how the combination of atomistic (aMD) and hybrid quantum/classical molecular dynamics (QM/MM MD) simulations with free energy (FE) sampling methods can be used to probe mechanistic principles of enzymes responsible for biological energy conversion. We present mechanistic explorations of long-range proton-coupled electron transfer (PCET) dynamics in the highly intricate respiratory chain enzyme Complex I, which functions as a redox-driven proton pump in bacterial and mitochondrial respiratory chains by catalyzing a 300 Å fully reversible PCET process. This process is initiated by a hydride (H^–^) transfer between NADH and FMN, followed by long-range (>100 Å) electron transfer along a wire of 8 FeS centers leading to a quinone biding site. The reduction of the quinone to quinol initiates dissociation of the latter to a second membrane-bound binding site, and triggers proton pumping across the membrane domain of complex I, in subunits up to 200 Å away from the active site. Our simulations across different size and time scales suggest that transient charge transfer reactions lead to changes in the internal hydration state of key regions, local electric fields, and the conformation of conserved ion pairs, which in turn modulate the dynamics of functional steps along the reaction cycle. Similar functional principles, which operate on much shorter length scales, are also found in some unrelated proteins, suggesting that enzymes may employ conserved principles in the catalysis of biological energy transduction processes.

## Key References

- RöpkeM.; SauraP.; RieplD.; PöverleinM. C.; KailaV. R. I.Functional Water Wires Catalyze Long-Range Proton Pumping in the Mammalian Respiratory Complex I. J. Am. Chem. Soc.2020, 142, 21758–217663332523810.1021/jacs.0c09209PMC7785131.^1^*First multiscale simulation study of the mammalian complex I, resolving proton pathways and QM/MM free energy profiles along key reaction steps*.
- RöpkeM.; RieplD.; SauraP.; Di LucaA.; MühlbauerM. E.; JussupowA.; Gamiz-HernandezA. P.; KailaV. R. I.Deactivation blocks proton pathways in the mitochondrial complex I. Proc. Natl. Acad. Sci. U. S. A.2021, 118, e20194981183427227510.1073/pnas.2019498118PMC8307655.^2^*Long-range coupling between conformational dynamics, proton transfer, and quinone oxidoreduction.*
- MühlbauerM. E.; SauraP.; NuberF.; Di LucaA.; FriedrichT.; KailaV. R. I.Water-Gated Proton Transfer Dynamics in Respiratory Complex I. J. Am. Chem. Soc.2020, 142, 13718–137283264337110.1021/jacs.0c02789PMC7659035.^3^*Link between ion-pair dynamics, proton transfer, and hydration changes in the bacterial Complex I.*
- Gamiz-HernandezA. P.; JussupowA.; JohanssonM. P.; KailaV. R. I.Terminal Electron-Proton Transfer Dynamics in the Quinone Reduction of Respiratory Complex I. J. Am. Chem. Soc.2017, 139, 16282–162882901732110.1021/jacs.7b08486PMC6300313.^4^*QM/MM exploration of the PCET mechanism linked to quinone oxidoreduction*.

## Introduction

Cellular respiration and photosynthesis are powered by membrane-bound enzymes that catalyze long-range proton-coupled electron transfer (PCET) processes, triggered by a chemical redox reaction or capturing of light quanta.^5^ The energy transduced via transient charge transfer states leads to the transport of protons across a ca. 30 Å thick lipid membrane (hydrophobic part), establishing a 100–200 mV electrochemical proton motive force (PMF) across the membrane. In analogy to the electromotive force in a battery, the PMF powers energy-requiring processes in the cell, such as active transport of solutes against concentration gradients and synthesis of adenosine triphosphate (ATP), the universal energy currency in biological systems.^6^

**Figure 1:**
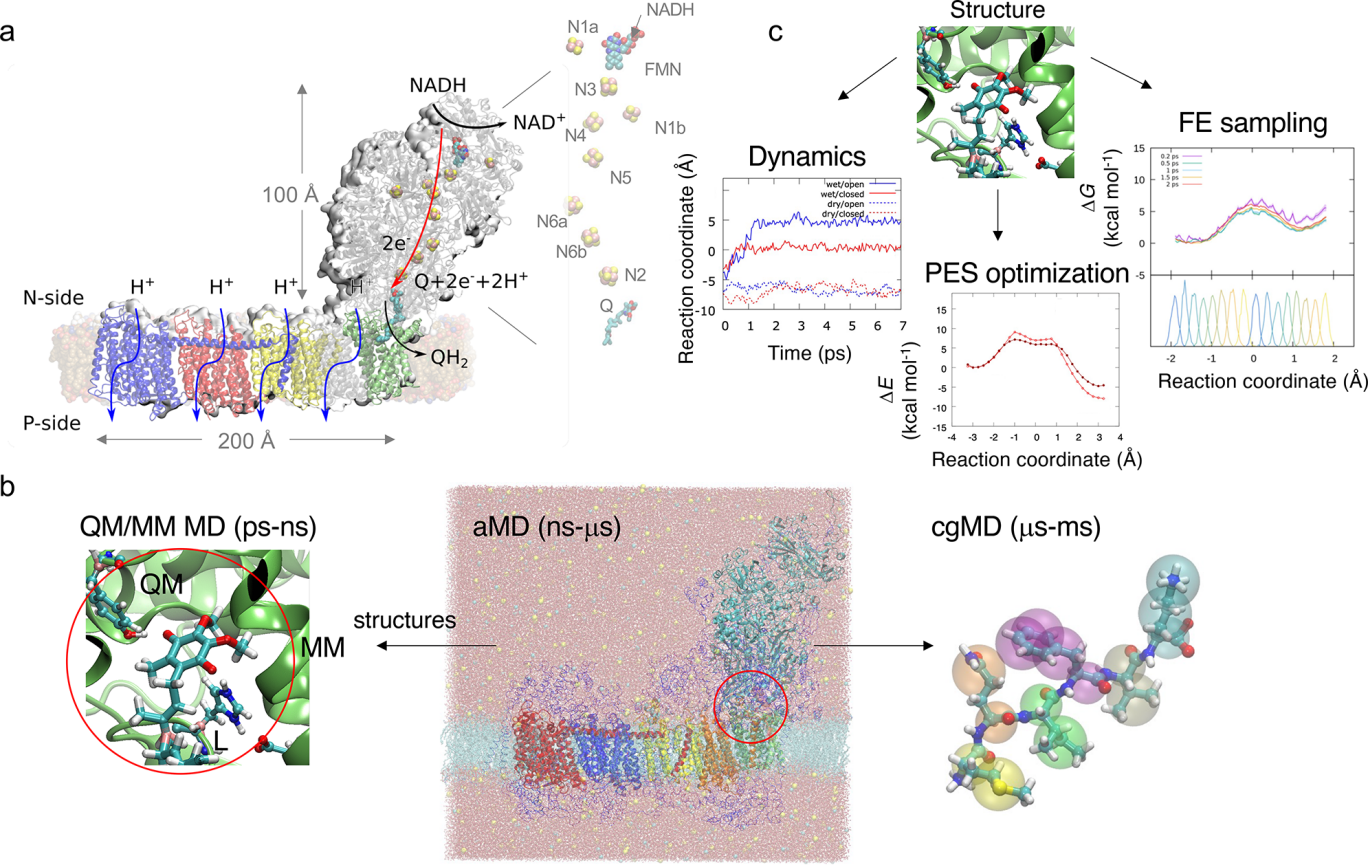
(a) Structure and function of Complex I. Reduced NADH donates electrons to the 100 Å long FeS chain that transfers them to quinone (Q). Q is reduced to quinol (QH_2_), which triggers the transfer of four protons across the 200 Å long membrane domain. Point mutations of residues in the proton pumping subunits (shown in blue, red, yellow, gray/green) lead to inhibition of the Q oxidoreduction activity. (b) Multiscale simulation approaches can be used for probing the structure, function, and dynamics of PCET mechanisms in Complex I and other energy transducing enzymes. QM/MM models (left) allow exploring the local electronic structure, energetics, and dynamics on picosecond time scales (QM, QM region; MM, MM region; L, link atom in pink); atomistic MD (aMD) simulations (middle) enable sampling of the microsecond dynamics in a model of the biochemical environment; whereas coarse-grained models (cgMD) (right, showing a 1:4 mapping of beads:heavy atoms) allow exploring the micro- to millisecond time scale, but with loss of atomic detail. (c) The systems can be explored by unbiased MD simulations, potential energy surface (PES) scans, or free energy sampling methods at the different theory levels. The MD simulations allow probing, e.g., the dynamics of a reaction coordinate over time (here proton transfer, PT), whereas PES scan or FE sampling allows computing free energy/energy profiles along a reaction coordinate of interest (here a PT reaction).

Understanding these key complex biochemical processes requires, in addition to knowledge of the exact molecular structures, methods that can resolve the dynamics of the system on catalytically relevant time scales. These time scales often extend several orders of magnitude, from the individual bond vibrations on femtosecond to picosecond time scales, to milliseconds, during which protons are released across the membrane in the respiratory and photosynthetic enzymes. While methods of structural biology, in particular, X-ray crystallography and cryo-electron microscopy (cryoEM), can resolve static structures of the protein complexes at an atomistic resolution, different spectroscopic techniques are often required to experimentally determine the dynamics along the biochemical reaction cycle. Exciting developments in time-resolved structural techniques have also been reported, particularly on light-triggered systems such as Photosystem II (PSII).^7^ Despite these impressive experimental developments, it still remains highly challenging to probe how and why a certain reaction intermediate results in given chemical or structural changes. To this end, multiscale simulations provide a powerful approach to computationally resolve the functional dynamics, energetics, and structural changes at a single molecule level and thus to probe molecular mechanisms in biological energy conversion. While quantum chemical (QM) methods are necessary to study the energetics and dynamics linked to chemical transformations, conformational changes coupled to the latter on a longer time scale require integration of more approximate atomistic molecular dynamics (aMD) simulations. The proteins responsible for biological energy conversion are often also very large, extending up to 1 MDa (∼150 000 atoms) as the mammalian respiratory Complex I, discussed herein (Figure 1a). An accurate computational model must, in addition to the dynamics of the protein itself, also predict the effect of the complex membrane/water/ion surroundings. The membrane as well as water molecules may modulate the protein dynamics and function, and properly accounting for these environmental effects may lead to large simulation models with up to a few million atoms (Figure 1b).

Here we describe the application of multiscale simulation approaches to bioenergetic enzymes with a focus on cellular respiration. We provide a brief overview of our recent work^1−4^ as well as both ongoing and future challenges in the modeling of Complex I (NADH: ubiquinone oxidoreductase), one of the most intricate redox-driven proton pumps known. For a more comprehensive review on Complex I, the reader should consult, e.g., ref (37) and references therein. Parallels are also drawn to other systems such as cytochrome *c* oxidase involved in cell respiration as well as Photosystem II, the light-driven water splitting enzyme that enables oxygenic photosynthesis. We start by briefly reviewing different simulation methods applicable to modeling these complex systems, followed by a description of the biochemical background and key mechanistic results. Notably, we use here the term PCET to describe *all* coupled proton- and electron-transfer processes, i.e., both stepwise and concerted reactions, and those within the immediate chemical bonds of the system as well as reactions extending across several hundreds of ångströms. The complete reaction catalyzed by Complex I comprises several substeps of individual local PCET, ET, and PT reactions. However, since these local steps are tightly coupled and the complete reaction is fully reversible, we characterize also the global 300 Å charge transfer (CT) reactions as a proton-*coupled* electron transfer process.

## Computational Approaches for Simulation of Biomolecular Dynamics in Energy Converting Enzymes

Computational treatment of chemical reactions and PCET/CT states involved in the respiratory and photosynthetic enzymes requires applications of QM methods that can accurately treat bond-breaking and bond-forming reactions, electronic ground and excited states, as well as the various spin/electronic states. These computational explorations can be achieved by (i) sampling the explicit dynamics of the system; (ii) by determining the potential energy landscape using reaction pathway methods; or (iii) by explicit dynamic sampling of the free energy (FE) surface, along a representative reaction coordinate/collective variable (Figure 1c). The dynamic exploration requires the computations of forces (and energies), which are used to propagate the dynamics of the system, often 1 fs at each time step (for [ground state] QM/MM MD; 2 fs common in aMD; 15–25 fs for cgMD). In contrast, different reaction pathway optimization methods (see, e.g., ref (8)) account for a restrained geometry optimization along the minimum potential energy surface (PES). While thermodynamic corrections along the PES can be included via computation of the molecular Hessian (second derivative of energy with respect to coordinate displacement, d^2^*E*/d*r*^2^), these approaches do not account for the dynamic sampling of the phase space. This can be important in biomolecular systems with low barriers along the FE landscape that may result in deviation from the minimum energy pathway. The FE surface can be explored in QM/MM simulations by umbrella sampling (US)^9^ or its string simulation variant,^10^ which involves restrained dynamic sampling of individual segments of the phase space. The FE profile (or potential of mean force) is then recovered by reweighting the individual windows based on the restraining potential employed, e.g., using the weighting histogram analysis method (WHAM).^11^ The application of QM/MM US methods is highlighted in the context of respiratory chain enzymes in the following sections. Although the dynamic sampling often relies on a classical integration of nuclear motion, biological CT/PCET reactions may also involve nuclear quantum effects, which must be treated using specialized computational techniques, but these will not be covered here. These effects often manifest in large (hydrogen/deuterium) kinetic isotope effects.^12^

In order to accurately describe the biochemical systems, hybrid quantum mechanics/classical mechanics (QM/MM) models are often applied,^13,14^ which allows for the treatment of both of the reactive QM system, embedded in explicit biological surroundings of the protein using classical atomistic force field models. QM/MM methods can be performed using mechanical, electrostatic, or polarizable embedding schemes that are implemented via additive or subtractive calculations of the total energy.^13,14^ In QM/MM methods with electrostatic embedding, commonly used in enzymatic applications, the QM region is explicitly polarized by the surrounding point charges, and forces arising from the QM system also affect the point charges of the MM region. Covalent bonds between the QM-MM boundary in the protein model can be modeled by the link atom approach, in which “dummy” hydrogen atoms are introduced between the side chain (Cβ) and backbone (Cα) atoms to saturate the valence of the QM subsystem, but which are not interacting with the MM region (Figure 1b). It is also possible to extend the regular QM/MM approach for the simultaneous treatment of multiple QM regions embedded in an MM surroundings that classically interact with each other.^14,15^ Moreover, the application of polarizable force fields has been shown to improve the accuracy of QM/MM, e.g., in the treatment of excited states of biochemical systems.^16^ However, the same effect may also be obtained by using large explicit QM regions, where the interactions beyond first protein residue neighbors are accounted for at the QM level.^17^

ET and PCET reactions require, in addition to an accurate electronic structure method, also the modeling of charge localized diabatic states, where the electron donor and acceptor groups undergo a redox change during the reaction process. While electronic structure methods normally aim to optimize the lowest energy electronic state, different charge localization schemes have been developed, such as the constrained DFT (cDFT) approach,^18^ which allows constraining the charge or spin of a given redox group to intermediate values (using a Lagrange multiplier, with the sum of spin/charge in the individual subsystems remaining constant).^18^ We note that multistate DFT methods^19^ may also provide new ways to systematically account for such PC/ET/CT reactions in bioenergetically relevant systems. The charge localized states can also be optimized by converging the molecular orbitals (MOs) of the individual reduced/oxidized fragments and then merging the fragment MOs into a specific combination of redox states.^4^ Particularly for PCET reactions, the ET step is often tightly coupled to a motion of a proton, the position of which can be used to control the redox state, and vice versa. Charge localized states can also be modeled by splitting the systems into different fragments: For example, via QM/MM methods, where the reduced/oxidized subsystems are modeled at the respective QM or MM levels, by multi-QM/MM methods, where simultaneous QM models as well as their redox states are included, or by frozen density embedding (FDE) methods,^20^ where the (frozen density) of the reduced/oxidized subsystem polarizes the active subsystem. The calculation of forces is still challenging at the FDE level, if covalent bonds are cut as in many biomolecular systems,^20^ thus limiting the applications of the method in MD applications.

The explicit dynamic sampling at the DFT level is on today’s processors and implementations often limited to 10–100 ps for systems with a few hundred QM atoms.^1−4^ This allows for the QM treatment of, e.g., the enzyme active site and its nearby first solvation sphere of surrounding amino acids. Similar to classical MD simulations, the development of GPU-accelerated QM codes in combination with different linear-scaling methods^21^ can further push these limits to longer time scales and larger systems. Semiempirical methods, such as tight-binding DFT methods (e.g., DFTB3^22^), or reactive force fields (e.g., the empirical valence bond (EVB) method^23^), allow via parametrization schemes for longer sampling time scales, and have therefore been applied for studies of several bioenergetically important systems.^24^ The PCET reaction in, e.g., Complex I can be highly challenging to accurately model with these more approximate methods, and to this end, first-principles based DFT-methods may provide a unique benefit despite their limited sampling capability. Nevertheless, the pico- to nanosecond sampling at both DFT and semiempirical levels, limits the direct exploration of reactions with rather low free energy barriers. This can in part be circumvented by initiating the QM/MM MD simulations from transient (nonequilibrium) states, obtained from snapshots of classical MD simulations, or by systematically exploring the FE-landscape using QM/MM MD sampling.

Lower-order correlated wave function based methods, such as the approximate coupled cluster theory (e.g., DLPNO–CCSD(T)^25^) or the random-phase approximation (RPA^26^), offer accurate ways to model electron correlation effects in CT reactions but still pose computational challenges for the explicit treatment of biomolecular dynamics due to their high computational costs (>*O*(*N*^5^), with the number of basis function, *N*). Nevertheless, correction of the PES or free energy surface can also be achieved at these levels, even for a few hundred atom systems, thus providing ways to account for systematic improvements in the electronic structure description.^1,2^

Linear-response time-dependent density functional theory (LR-TDDFT)^27^ is commonly used for the treatment of excited states in biomolecules, and is important for understanding, e.g., light-capturing mechanisms in photosynthetic systems. Lower-order coupled cluster methods, such as the second-order algebraic diagrammatic construction (ADC(2)) or the approximate coupled cluster theory, CC2, in combination with various approximations are also becoming feasible for extended biomolecular systems, such as large chlorophyll clusters in PSII (see, e.g., ref (28)), while CASSCF/CASPT2 methods have provided valuable methods for treatment of chromophores in several biochemical systems to account for multireference effects.^29^ While the latter models are still limited to the correlated treatment of a few tens of electrons, the development of, e.g., density matrix renormalization group (DMRG) approaches may further push these limits and provide future ways for the accurate treatment of multiple electronic states at an affordable computational cost, such as the highly complex electronic structure of the CaMn_4_O_5_ active center in Photosystem II.^30^

In contrast to the QM methods, which rely on a first-principles treatment of the electronic structure, highly parametrized atomistic biomolecular force fields can sample millions of atoms on several microsecond time scales at a low computational cost (Figure 1b) and thus provide a powerful approach to explore conformational and hydration changes linked to the PCET reactions. With the loss of atomistic detail but gain in sampling capacity, coarse-grained models (cgMD^31^) account for effective beads (e.g., by effectively mapping the interactions of four explicit atoms by one coarse grained bead, Figure 1b), allowing for the exploration of longer (μs to ms) time scales of bioenergetically relevant systems.^32,33^ Nonstandard transient charge separate states can be classically parametrized based on the QM or QM/MM calculations, which provides a more direct link between the QM/MM MD and aMD simulations. Biomolecular systems that undergo PCET/CT reactions may also change protonation states during the reaction. These processes can be highly challenging to model, particularly the charged buried networks in Complex I that contain hundreds of titratable residues (see below). Specialized techniques such as constant pH-MD in explicit solvent^34^ or multiconformation continuum electrostatic (MCCE) approaches^35^ can provide additional benefit to study such protonation effects. Development of polarizable force fields,^16^ where the charge distribution can polarize as a response to changes in the atomic positions, is likely to improve the description of charge separated states in MD, although these methods increase the computational cost relative to nonpolarizable force fields. Finally, we note that the development of machine learning methods, trained based on QM reference reactions,^36^ could further open up the simulation of large biochemical reactions at a low computational cost.

## Long-Range PCET Reactions in Complex I

The respiratory Complex I is one of the most intricate redox-driven pumps known in biology.^37,38^ It is a 0.5–1 MDa enzyme composed of 14–45 subunits embedded in the mitochondrial inner membrane or cytoplasmic membrane of bacteria. Complex I functions as an initial electron acceptor in aerobic respiration chains by oxidizing nicotinamide adenine dinucleotide (NADH), which originates from the metabolic breakdown of nutrients, and transferring the electrons via a protein-bound flavin mononucleotide (FMN) and a 100 Å chain of 8 iron–sulfur centers to ubiquinone (Q) (Figure 1a). The quinone is reduced in a 2e^–^ reduction step to ubiquinol (QH_2_) at the lower edge of the hydrophilic domain of the protein. The Q reduction and subsequent QH_2_ diffusion out from its binding pocket trigger proton pumping across the membrane domain of the protein, in subunits up to 200 Å away from the Q-active site (Figure 1a). Despite these large molecular dimensions, the PT across the membrane is tightly linked to the Q reduction and is fully reversible. The completed global reaction can thus be considered a long-range PCET reaction. Complex I can thus also use a ΔpH-gradient to oxidize quinol and transfer the electrons in the reverse direction along the ET chain.^39^ Consistently with the coupled nature of the ET and PT, mutations of distant parts of the terminal proton pumping subunits leads to a measurable effect in the Q oxidoreductase activity.^3,40^ In addition to the physicochemical importance, the enzyme is also biomedically highly significant as nearly half of all human mitochondrial disorders are linked to point mutations in Complex I.^38^ Remarkably, homologues of Complex I, such as the “photosynthetic Complex I” variant, enable cyanobacteria to concentrate CO_2_, most likely driven by the same redox-driven proton pumping machinery as in the canonical variant.^68^ However, despite several resolved structures and biophysical experiments, the long-range PCET mechanism and its energy transduction principles still remain unclear.

Our research group has studied the puzzling PCET mechanism of Complex I by various multiscale computational methods that provide a powerful approach to probe the functional dynamics in key reaction steps, but also valuable input for designing new experiments. The combination of computational, structural, biochemical, and biophysical approaches in recent years has unraveled several key principles of this fascinating enzyme.

### Initial PCET Reactions Linked to NADH Oxidation and Long-Range Electron Transport

**Figure 2:**
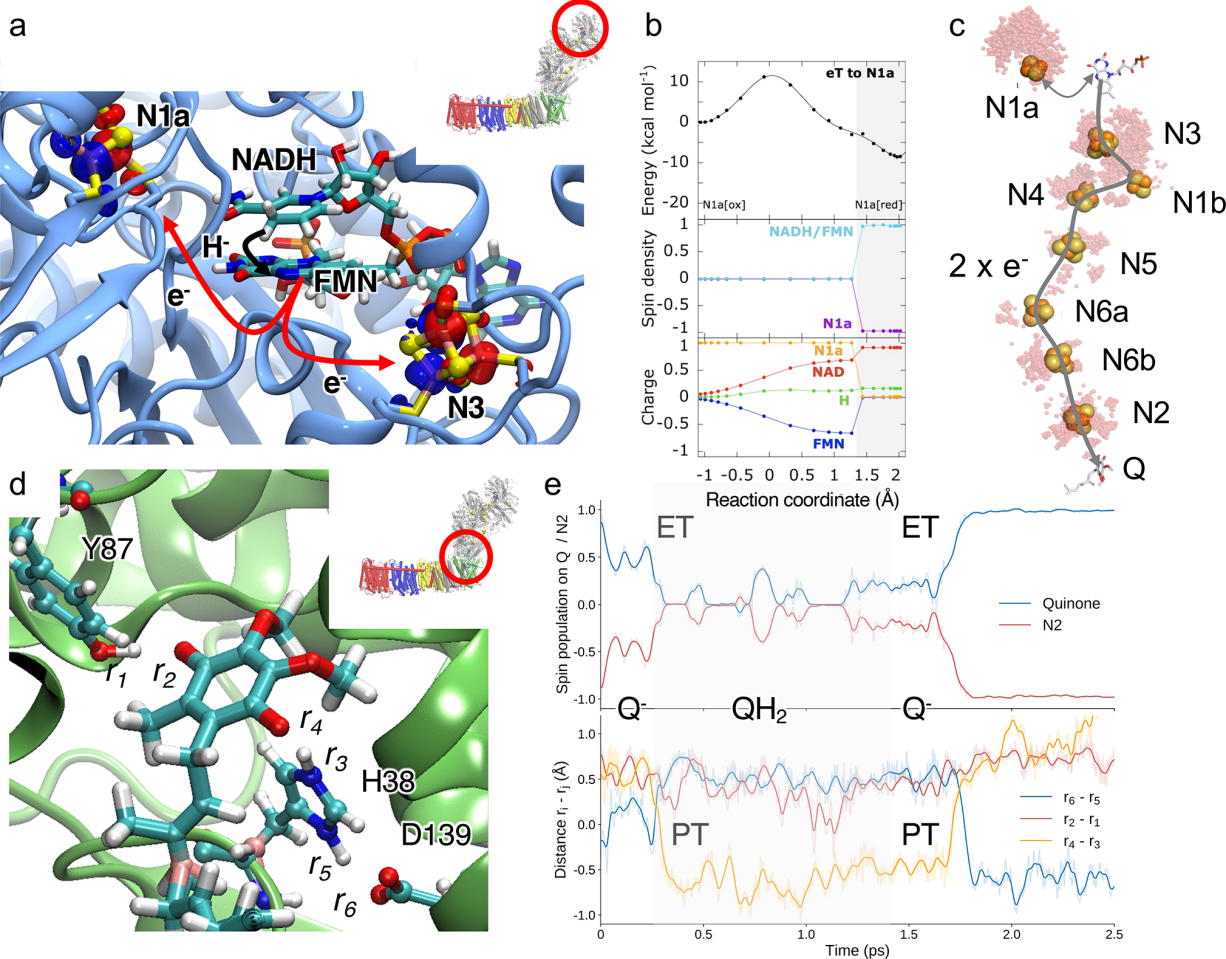
Multiscale simulations of PCET reactions in Complex I. (a) PCET between NADH and FMN leads to hydride (H^–^) transfer between the cofactors, followed by ET to the nearby FeS centers. (b) Energy profiles, spin, and charge analysis of the cofactors along the PCET process and coupled ET (here to N1a) along a proton transfer reaction coordinate. All FeS center were initially modeled in their oxidized state. The change in redox state of N1a is indicated in the top panel. (c) ET pathway down along the 100 Å FeS chain to Q. The figure also shows water molecules surrounding the FeS clusters. (d) QM/MM model of the Q oxidoreduction process: PCET between the terminal N2 FeS and Q triggers PT from nearby Tyr and His residues. (e) QM/MM MD of the PCET process shows the reversible formation of the QH_2_ by ET from the reduced N2 FeS center, and vice versa. Reproduced from refs (4) and (41). Copyright 2017 and 2019, respectively, American Chemical Society.

The hydrophilic domain of Complex I catalyzes the initial ET reaction from NADH to Q. This process includes three functional “units” in the canonical enzyme: the NADH/FMN site, a chain of 8 FeS centers, and the Q oxidoreduction site (Figure 1a). The reduced NADH docks to the FMN at the top edge of the hydrophilic domain of Complex I by dispersive van der Waals interactions (Figure 2a). The close (∼3.5 Å) contact between the aromatic ring systems^41,42^ enables hydride atom (H^–^) transfer between the cofactors (Figure 2a,b) with a free energy barrier of around 10–12 kcal mol^–1^ (at the B3LYP-D3/def2-TZVP/MM level),^41^ which compares well to experimentally determined *k*_cat_ < 15 000 s^–1^.^43^ Both experimental and computational estimates of the H/D kinetic isotope effects are in the range of 4 (modeled at the DFT level based on shifts in zero point vibrational effects), suggesting that no large nuclear tunneling effects are involved in the PCET process.^41^ The resulting reduced flavine (FMNH^–^) triggers further ET to two nearby FeS centers, the binuclear N1a and the tetranuclear N3 (Figure 2a,b), at least in the bacterial variants of the enzyme. Reduction of the N1a FeS cluster enhances the binding affinity of NAD^+^ by subtle conformational changes of charged residues in the binding pocket.^41,44^ The NAD^+^ is likely to stay bound until the electrons are transferred down along the FeS chain, a process that could prevent the formation of reactive oxygen species (ROS).^37−39^

The electrons then continue, one at the time, via the FeS chain, with individual centers separated by ca. 8–14 Å (*edge*-*to*-*edge*) distance from each other, to Q, which binds in a narrow pocket, ca. 20 Å above the membrane plane (Figure 2c). Electrochemical experiments,^45^ as well as DFT calculations,^4^ which allows determining redox potentials as well as other electron transfer parameters based on the electronic structure,^37^ suggest that the FeS are nearly equipotential to each other. The exception is the terminal N2 center, which has a somewhat higher redox potential.^4,43^ EPR-freeze quench experiments^43^ show the ET takes place on ca. 90 μs time scales through the FeS chain, whereas similar ET time scales are also predicted based on tunneling pathway analysis,^46^ or by applying nonadiabatic electron transfer theory with typical distance dependent electronic couplings, reorganization energies in the 0.3–0.7 eV range, and thermodynamic driving forces Δ*G* in the 0–200 mV range.^37,43^ This suggests that the long-range ET process itself is unlikely to link to protonation- or large-scale conformational changes, which would be expected to result in longer transfer time scales. These observations also suggest that the proton pump is activated after the long-range ET, i.e., upon or after the Q reduction event.^37^

On the electronic structure level, the tetranuclear (N3, N4, N5, N6a, N6b, N2, Figure 2c) FeS centers undergo redox changes between the 2Fe^III^2Fe^II^/1Fe^III^3Fe^II^ forms, whereas the binuclear N1a, N1b (Figure 2c) change between the Fe^II^Fe^II^/Fe^III^Fe^II^ forms. Each of the FeS centers contain four to five unpaired electrons, which are antiferromagnetically coupled together, yielding the *S* = 1/2 or S = 0 state for the reduced and oxidized cluster, respectively. The six unique spin configurations of each redox state in the tetranuclear clusters (two spin configuration for the binuclear clusters) can be computationally modeled using, e.g., broken-symmetry DFT.^47^ Accurate treatment of the spin energetics for these clusters may also require methods that can treat electron correlation effects from first-principles (see above). Interestingly, DFT calculations suggest that many of the different spin configurations are nearly isoenergetic in Complex I, although the center of the charge distribution in these states varies within the clusters. As the electronic coupling is strongly distance dependent (*H*_ab_ ∝ *e*^–*r*^), switching between the different spin states may contribute to the overall effective ET down the FeS chain.

Interestingly, the stepwise reduction of the FeS centers leads in aMD simulations to a transient increase in the hydration around the clusters (Figure 2c) as well as subtle conformational changes of charged residues surrounding the clusters,^1,41^ which affect both redox potentials and reorganization energies for the ET process but do not seem to change the nonadiabatic nature of the ET process. The hydrophilic domain has also been found to undergo a bending and twisting motion on microseconds time scales,^1,2,37,50^ which are indirectly supported by cryoEM classification of different structures.^48,49^ Based on simulation studies,^32,50,51^ this motion has been suggested to support the Q diffusion and binding to its binding site (see below).

### Quinone Reduction Triggers Conformational Changes

**Figure 3:**
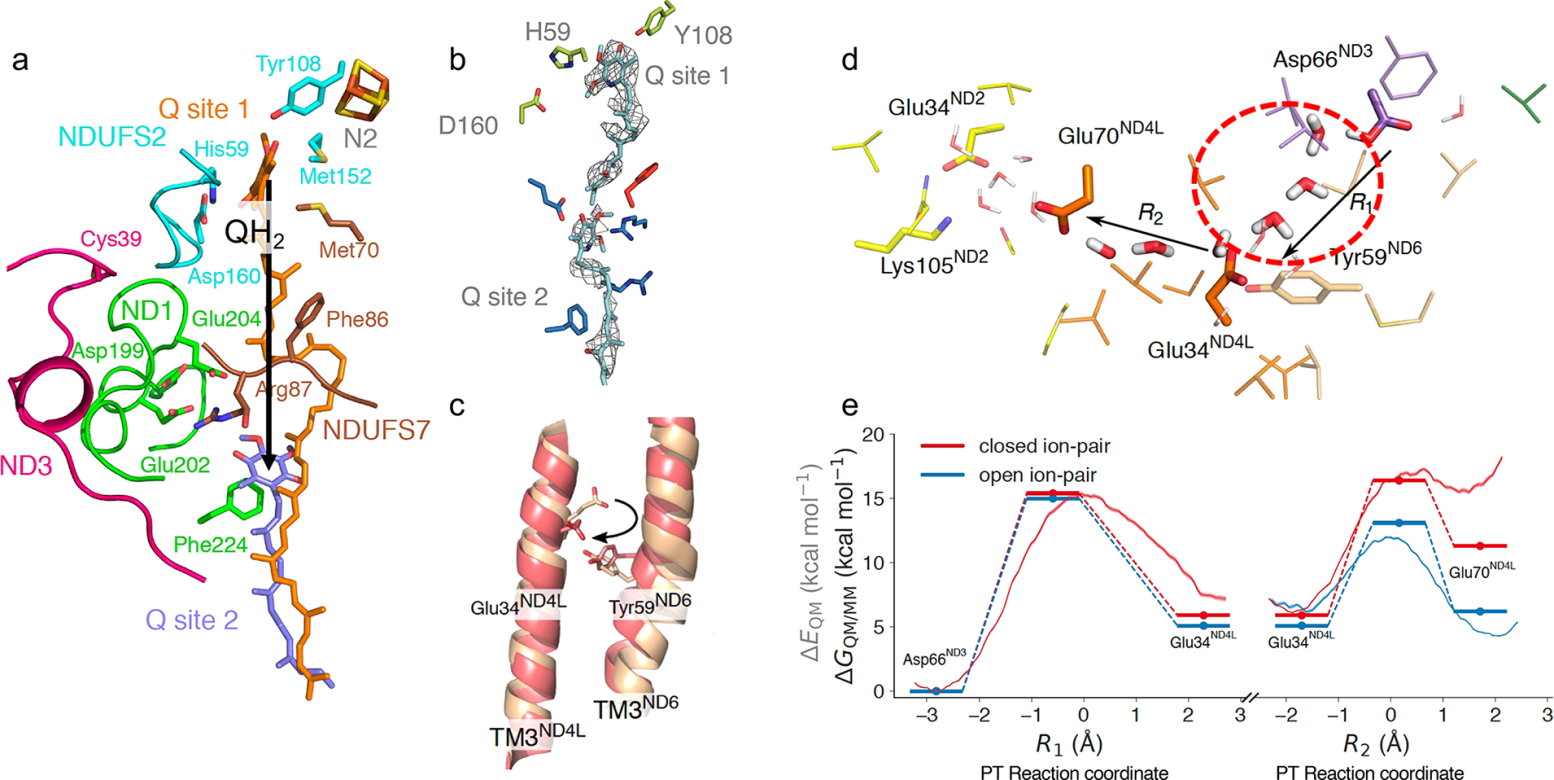
(a) Quinol formation in the active site (site 1) leads to dissociation of the QH_2_ to a second binding region (site 2). The process is coupled to conformational changes in charged residues and surrounding loop regions. (b) CryoEM map for piericidin A and modeled end-on binding mode of two inhibitors bound in the Q-cavity. Data from ref (55). (c) Conformational changes between transmembrane helices (e.g., TM3 of ND6) in the membrane domain of Complex I regulate the formation of (d) proton conducting water wires (marked in red circle). (e) Free energy profiles computed based on QM/MM US simulations (profile) and QM cluster models (energy levels based on reaction pathway optimization) for the PT from the E-channel region to residues at the ND2 interface. The amino acid binding the proton is marked in the free energy profile. *R*_1_ and *R*_2_ are reaction coordinates used to model the PT reactions (see ref (2) for further details). Reproduced with permission from ref (2). Copyright 2021 the Authors. Published by PNAS. This open access article is distributed under Creative Commons Attribution-NonCommercial-NoDerivatives License 4.0 https://creativecommons.org/licenses/by-nc-nd/4.0/.

QM/MM MD and aMD simulations suggest that Q is stabilized by hydrogen-bonding interactions with Tyr87 (*Thermus thermophilus* numbering, Tyr108 in the mammalian enzyme, Figure 2d),^4,52^ a highly conserved residue in the quinone binding site, whereas both hydrogen-bonded and stacked interactions are formed between the Q and His38 (His59 in mammals).^4,55^ The long isoprenoid tail of ubiquinone (Q_10_) forms several nonspecific interactions with residues along the ca. 40 Å long L-shaped substrate channel.^53^ When quinone or semiquinone (SQ, formed by a one-electron reduction of Q) is present, His38 favors the protonated imidazolium (HisH^+^) state, which forms an ion pair with the nearby Asp139 (Asp160 in mammals). The SQ, which remains anionic (Q^•/–^) in the active site during QM/MM MD simulations,^4^ is likely to be transient and does not accumulate during turnover.^54^ The computationally predicted binding modes of Q have recently gained experimental support from cryoEM structures of inhibitor-bound forms of Complex I (Figure 3b).^49,55,57^ During a second ET step from the terminal N2 FeS center to the SQ, Tyr87 and His38 function as immediate proton donors for the reduced quinone, forming a quinol species (QH_2_) within the active site (Figure 3e).^4^ PT from His38 to the Q weakens the ion pair between the histidine and Asp139, resulting in subsequent conformational changes in residue side chains and surrounding loops that propagates along a chain of charged residues toward the membrane domain of Complex I.^52,56^ These conformational changes may link to proton uptake in the membrane domain, as indirectly supported by mutagenesis experiments.^52^ The PCET reaction coupled to quinol formation (*E*_m,7_ = −320 to −260 mV) could be nearly isoenergetic with that of the NADH oxidation (*E*_m,7_ = −320 mV).^4,37,45^ This indicates that the QH_2_ formation does not result in an immediate large energy transduction step, which is rather expected to take place upon diffusion of quinol toward the membrane, where quinones are at higher potentials (*E*_m,7_ ∼ +90 mV).^37^ At a computational level, these reactions involve long-range CT processes that can be challenging for DFT methods and may require application of higher order theory, such as RPA. RPA has been applied to study some of the long-range PT reactions in Complex I.^1,2^

Upon formation of the QH_2_ and dissociation of the nearby His/Asp ion pair, free energy explorations suggest that the quinol diffuses along its long cavity toward a second membrane-bound binding site (Figure 3a),^56^ the location of which has now been supported by recent structural and functional experiments (Figure 3b).^49,55,57^ At this second binding region, aMD simulations show that the quinol can form contacts with several charged and aromatic residues, as well as with a chain of conserved carboxylates within the so-called E-channel region (due to the many glutamates). The surroundings may stabilize the formation of an anionic quinol (QH^–^) species via PT to one of the glutamates^2^ that, in turn, could initiate a long-range proton pumping cascade along the membrane domain (see below). The p*K*_a_’s of the buried carboxylates at this region are highly shifted from their solution values based on QM/MM as well as electrostatic calculations,^2^ although the computational treatment of the exact titration behavior is highly challenging. Interestingly, recent spectroscopic data provide possible further support for the involvement of QH^–^ species.^58^ The Q reduction and diffusion to the second binding site have also been found to link to conformational changes in several dynamically flexible loop regions surrounding the subunit (Figure 3a) that are partially explored during microsecond aMD simulations as well as millisecond cgMD simulations.^2,32^

It is also worth noting that while the discussed computational methods have their unique power in probing complex biochemical processes, they also have their limitations. For example, it is highly challenging to account for long-time scale dynamics in QM and QM/MM simulations and chemical reactivity or polarization effects with aMD methods, while molecular details and quantitative interactions cannot always be accurately captured with cgMD methods. The power in combining simulations across multiple scales provides unique benefits in modeling the highly intricate PCET reactions in Complex I, which has an overall turnover in the millisecond time scale, but elementary processes most likely taking place on much faster, pico- to nanosecond time scales. Combinations of computational methods, together with structural, biochemical, and biophysical experiments are thus needed to elucidate the unknown mechanistic principles of this highly challenging system.

### Conformational Gating and Initiation of the Long-Range Proton Transfer Cascade

**Figure 4:**
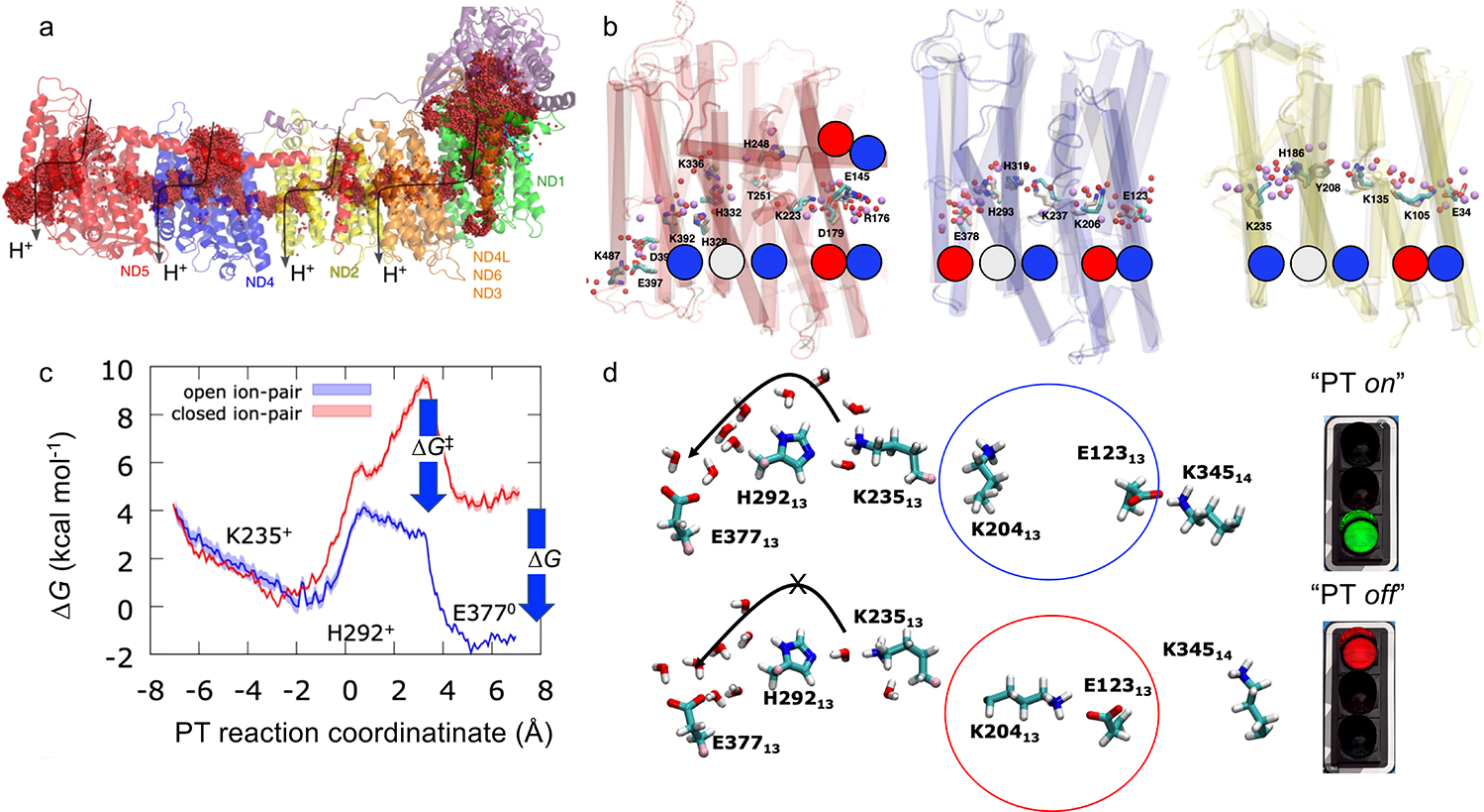
(a) Microsecond MD simulations predict S-shaped proton pathways across the membrane (averaged water structure). (b) Comparison of the water wires based on MD simulations (water in red spheres) and cryoEM experiments (in purple). Data from ref (61). Structure of the charged array along the center of the membrane domain is highlighted with Lys(Arg) (spheres in blue) and Glu(Asp) (in red), and His (in gray). (c) QM/MM free energy calculations of PT along a predicted water wire in the antiporter-like subunits with open and closed ion pairs. The PT reaction coordinate is defined as a linear combination of bond-forming and bond-breaking distances along the PT reaction from K235 to E377 (see ref (3) for further details). (d) Ion pair (IP) opening enables lateral PT within the antiporter-like subunits. Protonation of the terminal residue favors opening of the IP in the next subunit. Green signal, favorable PT; red signal, unfavorable PT. Adapted from refs (1) and (3). Copyright 2020 American Chemical Society.

The membrane domain of Complex I undergoes specific hydration changes that establish hydrogen-bonded water arrays connecting the negatively charged side (N-side) of the membrane with buried parts of the membrane domain and further across the membrane to the positively charged side (P-side) (Figure 4a). aMD simulations have provided powerful ways to predict the structure of these proton wires,^1−3,37,59,60^ that are now also starting to become resolved in cryoEM structures (Figure 4b).^49,61^ The combination of the aMD simulations with QM/MM-MD and QM/MM FE explorations has further allowed the exploration of the long-range PT dynamics within these proton wires (Figure 4c, d).^1−3,37,59^

Recent work on the mammalian Complex I also revealed conformational changes in a transmembrane helix (TM3) of subunit ND6, which is located close to the second membrane-bound Q binding site, and undergoes a rotation between α-helical and π-bulge forms.^2,48,49^ This conformational change, that we have explored by aMD simulations,^2^ allows for the formation of a hydrogen-bonded water array between the carboxylate chain near the membrane-bound Q site and the antiporter-like subunits,^2^ which support PT across the region (Figure 3c–e). In contrast, the closed state of this gating region seems to block the propagation of the protonation signal from the second Q site toward the other proton-pumping subunits (see below).^2^ Hydration changes at this region is also supported by recent cryoEM structures.^49^ These conformational changes could be of relevance in blocking the coupling between the PT and ET reactions during Complex I deactivation, e.g., by preventing the consumption of ΔpH-gradient to oxidize quinol and catalyze reverse ET up the FeS chain.^2^ This could provide a protective function upon rapidly changing respiratory conditions, e.g., in hypoxia caused by stroke,^2^ during which the respiratory chain reverses its direction and Complex I produces reactive oxygen species (ROS).^39^

### Proton Pumping via Electrostatic Cradle Mechanism

The membrane domain of Complex I comprises the antiporter-like subunits, ND2 (in mammals Nqo14 in *T. thermophilus*), ND4 (Nqo13), and ND5 (Nqo12) that contain a chain of charged residues along the central hydrophilic axis of the subunits. Molecular simulations show that these chains establish S-shaped water-mediated proton pathways across the membrane (Figure 4a),^37,59,60^ that are regulated by the protonation state of the ionizable residues themselves.^3,59^ The computationally predicted hydration structure^1−3,59,68^ has recently been supported by improved resolution in cryoEM structures (Figure 4b).^49,61^

**Figure 5:**
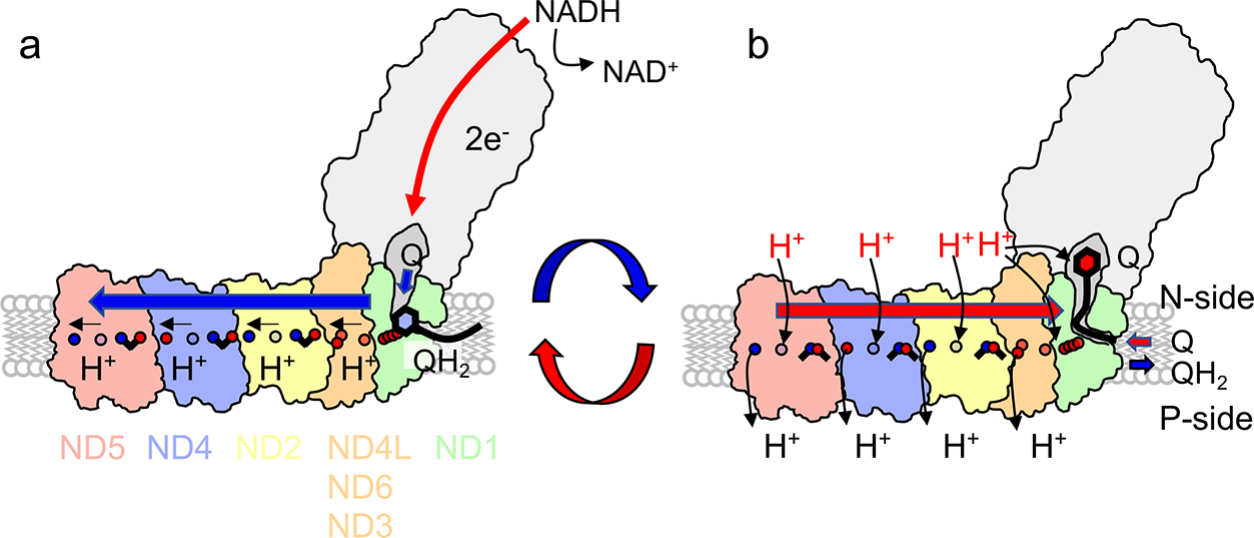
Schematic representation of the putative long-range PCETmechanism in Complex I. (a) NADH-driven ET along the FeS chain reduces Q to QH_2_, which triggers conformational changes and motion of the QH_2_ to a second binding site. The QH_2_/QH^–^ initiate stepwise PT reactions that lead to consecutive opening/conformational changes of ion pairs, and modulate the energetics of lateral PT reactions. The signal propagates to the terminal edge of ND5 (in red). (b) Proton release across the membrane increases the p*K*_a_ of the middle Lys, leading to H^+^ uptake from the bulk (H^+^ in red) and closure of the IP in the last subunit (subunit in red). The closed IP destabilizes the proton stored at the ND4/ND5 interface that is ejected across the membrane. The signal propagates “backward” in a similar way via ND4 (in blue), ND2 (in yellow), and ND4L/ND1(orange/green) to the quinol, which is ejected to the membrane. The new reaction cycle is initiated by reprotonation of the Q-active site and uptake of a new Q from the membrane. Adapted from ref (3). Copyright 2020 American Chemical Society.

Each antiporter-like subunit also comprises a conserved ion pair (IP) in the vicinity of the chain of the ionizable residues (Figure 4d). Simulations suggest that in the closed conformation of the IP, the “middle” lysine residue favors a protonated state, whereas opening of the IP lowers the free energy barrier and increases the driving force for lateral PT along the chain (Figure 4c).^3^ The protonation of the last element in each antiporter-like subunit favors opening of the next IP in the chain. A similar effect would also be achieved by PT between the subunits, although high free energy barriers may block the process.^1^ The stepwise IP opening and lateral PT steps result in a charge propagation cascade toward the end of the last subunit in Nqo12 (ND5) (Figure 5a). The proton released across this terminal subunit, which forms a hydrated contact to the P-side bulk,^37,59,62^ increase the proton affinity of the middle lysine, which in turn destabilizes the open state of the IP within the same subunits.^37^ Upon switching to the closed state of the IP, the proton stored at the interface region is ejected to the P-side, followed by protonation and closing of the IP with ND4 (Figure 5b). Similar events propagate to ND2 and finally toward the Q-second binding region, where the PT via the putative E-channel region, could release the quinol to the membrane (Figure 5b).

Our electrostatic forward/backward wave model resembles a Newton’s cradle device,^1,3,37^ in which a mechanical propagation swings in a forward and backward motion (Figure 5). This also implies that the removal of the terminal subunits leads to backpropagation of the signal from the penultimate subunit and reduction of the overall proton pumping stoichiometry, consistent with experimental observations.^63^ In contrast, it was recently suggested,^49^ based on a few resolved water molecules at the P-side exit pathway of the ND5 subunit, but not at the other subunit interfaces, that all four protons could be pumped across the terminal edge of ND5. However, this ND5-only pumping model speaks against the experimental evidence, where proton pumping is still observed despite the removal of terminal subunits.^63^ Moreover, similar to many other proton conducting regions, e.g., in cytochrome *c* oxidase, transient but functionally central water molecules are not always structurally resolved even at high resolution.^64^

### Concluding Remarks and Mechanistic Comparison to Other Energy Transducing Enzymes

The long-range PCET mechanism in Complex I, described in this Account, is suggested to involve the following key steps (Figures 4 and 5): (i) The long-range ET-driven quinone reduction to quinol triggers conformational and electrostatic changes in charged residues around the active site and surrounding regions (Figure 2d, e). (ii) The motion of the quinol to a second binding region (Figure 3a) triggers lateral PT reactions via the formation of water-wires (Figure 3c–e), controlled by the protonation state of ionizable residues in the proton channels themselves. (iii) Protonation of conserved residues at the interface of antiporter-like subunits leads to the conformational change of a buried IP in ND2, which (iv) modulates the PT barrier and enables lateral PT reactions along the same antiporter-like subunits (Figure 4c, d). These PT reactions are enabled by the hydration of the channels that, in turn, are strongly coupled to the protonation state of the ionizable residues. (v) The “protonation signal” propagates across the three antiporter-like subunits, ND2, ND4, and finally to terminal ND5 (Figure 5). (vi) Proton release across the membrane in ND5 triggers a backward propagation of the PT signal, which leads to (vii) subsequent closing of the IP and (viii) uptake of protons within the same antiporter-like subunits. The process propagates via ND5 to ND4, and ND2 to the membrane-bound Q binding region. (ix) Reprotonation of the “queuing” QH^–^ in the second binding site could release it as a quinol and (x) initiate a new reaction cycle. Due to microscopic reversibility, this mechanism is also expected to work in reverse, enabling the ΔpH-driven quinol oxidation process.

**Figure 6:**
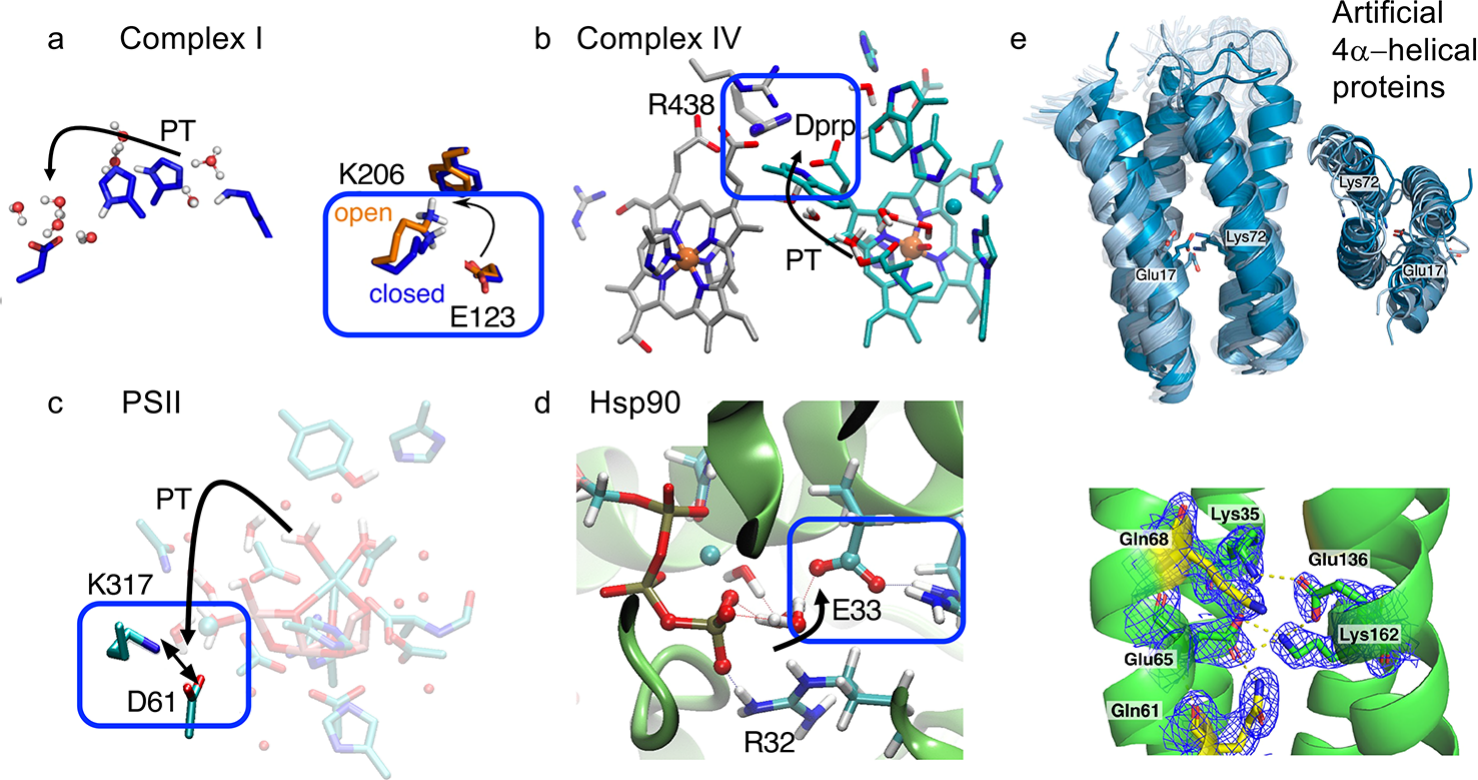
Comparison of functional elements involved in CT/PCET reaction in different enzymes. (a) Complex I (closeup of antiporter-like subunit ND4). (b) Cytochrome *c* oxidase (nonpolar cavity around the active site). (c) Photosystem II (vicinity of the CaMn_4_O_5_ cluster). (d) ATPase reaction in Hsp90 (active site in the N-terminal domain) (see main text). (e) Designed buried ion pairs in artificial bundle proteins, showing different ion-pair conformations, with aims to understand functional principles from a bottom-up approach. All ion pairs shown are located in buried core regions of the proteins. Panel (a) adapted from ref (1). Copyright 2020 American Chemical Society. Panel (e) adapted with permission from ref (67). Copyright 2021 the Authors. Published by Springer Nature under the terms of the Creative Commons Attribution License http://creativecommons.org/licenses/by/4.0/.

Similar functional motifs are also observed in several other bioenergetically relevant systems: In cytochrome *c* oxidase (C*c*O), the terminal enzyme of the respiratory chain, the reduction of an electron-queuing heme opens up an IP between the heme propionate group and arginine residue (Figure 6b).^64^ This could lead to electric field variations, similar to the IP opening in Complex I (Figure 6a), and result in the modulation of the PT energetics to a so-called pump site, whereas reduction of the active site seems to lower PT barriers by field effects to the latter.^64,65^ Conformational changes in IPs (e.g., the Lys317/Asp61) could also modulate redox-driven PT barriers from the oxygen-evolving CaMn_4_O_5_ center to residues participating in proton release in Photosystem II (Figure 6c).^69^ Similar electrostatic effects have also recently been described in the molecular chaperone Hsp90 (Figure 6d), where an IP dissociation (Glu33/Arg32) modulates the reaction barrier for ATP hydrolysis^66^ and which further triggers large-scale conformational changes in the chaperone structure. The conservation of such functional elements has recently inspired us to design buried ion pairs using artificial minimal protein frameworks,^67^ that could help to dissect and probe the conformational dynamics and intriguing mechanistic principles in simplified biochemical model systems from a bottom-up approach (Figure 6e).

In conclusion, the integration of multiscale simulations studies with biochemical, biophysical, and structural approaches have provided unique ways to unravel mechanistic principles of PCET reactions governing biological energy transduction mechanisms. The development of new computational methods in combination with the recent structural revolution in biochemistry will further increase the complementarity between theory, simulations, and experiments, eventually allowing us to understand molecular principles of the highly intricate PCET mechanisms in Complex I as well as in other remarkable enzymes.
